# The existential is essential

**DOI:** 10.1192/j.eurpsy.2026.10279

**Published:** 2026-07-31

**Authors:** M. Calmeyn

**Affiliations:** private practice ‘Lelieveld’, Loppem Zedelgem, Belgium

## Abstract

**Abstract:**

In the beginning of 2026 the Superior Health Council published an advice on the psychosocial well-being in residential care centers. The ‘usual suspects’ on this subject as education, medicalization, innovation and internal organization are evaluated and given new incentives. It soon became clear that the existential is not just another box that needs ticking. On the contrary, it permeates the whole project of living well and well being in general. In residential care centers moreover people are confronted with frailty but also with (renewed) resilience. The importance and implementation of the existential dimension in the centers will be presented and discussed.

**Disclosure of Interest:**

None Declared

